# A questionnaire-based study of Paediatric Dentists’ knowledge of teething signs, symptoms and management

**DOI:** 10.1038/s41405-022-00099-4

**Published:** 2022-03-12

**Authors:** Lauren Reeve-Brook, Shannu Bhatia, Waraf Al-Yaseen, Nicola Innes, Nigel Monaghan

**Affiliations:** 1 University Dental Hospital, Heath Park, Cardiff, CF14 4XY United Kingdom; 2 School of Dentistry, College of Biological and Life Sciences, Cardiff University, Heath Park, Cardiff, CF14 4XY United Kingdom; 3 Public Health Wales, Temple of Peace and Health, Cathays Park, Cardiff, CF10 3NW United Kingdom

**Keywords:** Paediatric dentistry, Oral conditions

## Abstract

**Introduction:**

Signs and symptoms attributed to erupting primary teeth are commonly known as “teething”. Its existence is controversial with concern that overusing this term might mask other illnesses and some treatments may be unnecessary or harmful. Parents/carers can access over-the-counter remedies and may seek professional advice. This survey-based investigation explored gaps in knowledge, training, perceptions and experiences of UK Paediatric Dentists (PDs) regarding teething in young children.

**Method:**

Cross-sectional study with a convenience sample of PDs with different training levels, accessed through the British Society for Paediatric Dentistry membership lists. A 10-item questionnaire explored participants’ knowledge of teething-related issues and management. Responses are presented using descriptive statistics.

**Results:**

Of 80 responding PDs (response rate 27%), 62–74% suggested drooling, irritability, oral fixation and flushed cheeks were attributed to primary tooth eruption. Fifty-eight (72%) participants were unaware of guidelines, yet 53 (66%) felt guidance was needed.

**Conclusion:**

Signs and symptoms of teething might mask underlying conditions so it should be a diagnosis of exclusion. PDs attributed similar signs and symptoms to teething, as have been reported in the literature, although some commented that they did not think teething was a condition. There was a lack of awareness over guidelines.

## Introduction

There are numerous surveys and observational studies from around the world [1–5] showing that parents strongly associate a long list of signs and symptoms with tooth eruption, commonly known as “teething”. However, there is ongoing debate as to whether these are simply a manifestation of an association between primary tooth eruption and the period of time when children’s maternal immunity starts to recede and they begin to move and explore their environment, both of which present a challenge to the immune system and increasing likelihood of infection [6, 7].

The eruption of a child’s first primary tooth is often seen by parents, wider family and carers as a milestone event, beginning when babies are around six months and complete when the child, now a toddler, has an established primary dentition and is two to three years of age. It is a physiological process that lasts for years, with periods of activity and inactivity. Reports of teething and its signs and symptoms are limited to babies and young children with no evidence of these systemic signs and symptoms being present in older children/ adolescents. Systematic reviews [7, 8] have attempted to determine the prevalence of teething, the most commonly reported signs and symptoms and whether there is any association between them and tooth eruption. The prevalence of teething in 0–36-month-old children has been reported as 71% [9] with gingival irritation, irritability and drooling the three most common signs and symptoms but loss of appetite, oral fixation, sleeping problems, rhinorrhoea, fever, diarrhoea, rash, vomiting and others also recorded. One systematic review specifically looking at the relationship between fever and tooth eruption, found an association with fever only when a rectal temperature was taken [10]. The National Institute for Health and Care Excellence (NICE) [11] advises exclusion of other conditions before making a diagnosis of teething, as more serious conditions could be overlooked, particularly if the infant is “systemically unwell and showing signs of severe distress”.

In terms of dental health care practitioners’ views about teething, there was no evidence from the UK. However, there is evidence from other countries (the US and Nigeria) that suggests dentists often believe teething to be an independent problem that can cause fever and diarrhea [12, 13]. Furthermore, Oziegbethis et al in 2011 [12], found that dentists who shared these beliefs, acted upon them despite their awareness that they were culturally acquired through their personal experiences, school, and myths, rather than being derived from science or evidence.

To reduce the distress for the child associated with these signs and symptoms attributed to teething, a variety of pharmacological and non-pharmacological “teething remedies” are available that can be “home” remedies or available over the counter (OTC). OTC medicines do not need a prescription and can be obtained directly from a supermarket or pharmacy and without the advice of a dental or allied health professional (AHP) [14]. These can include systemic or topical analgesics, antibiotics or other chemicals [15]. More culturally centred remedies include amber teething necklaces, gum incision [16], applying herbs to the gums, and the use of rehydration salts and vitamins amongst many others. Quaternary prevention (the avoidance of over medicalisation and treatments that might have more harm than benefit) [2, 17] should be a constant in guiding treatment recommendations. Some teething treatments involve medicaments (such as topical anaesthesia), are unproven and might be unnecessary [18], the active components and potential adverse effects of some are unknown, and others have caused significant harm [19]. A recent review of the evidence in the British Dental Journal [20], found 14 licenced teething products where there was limited evidence of benefit and of which 9 were potentially harmful (containing sucrose or chemicals such as alcohol or lidocaine. Paediatric dentists (PDs) are expected to have good understanding of teething because of their level of training and clinical exposure to child patients, however little is known about their teething-related knowledge, resource use or the advice they give to parents, general dentists, or other AHPs. Some evidence from other countries such as the United Stated, have suggest PD believed that teething is associated with diarrhoea and other symptoms such as swollen gums, drooling, irritability, inflamed gums, restlessness, sleeplessness, and fever and

## Aims and objectives

The aim of this survey-based investigation was to explore gaps in knowledge, training, perceptions and experiences of PDs and Paediatric Dentistry Speciality Trainees in the UK in relation to teething with the specific objectives being to investigate:

1. Signs and symptoms considered to be most commonly attributed with teething;
2. Participants’ perceived undergraduate education around teething;
3. The frequency with which participants have to provide advice for teething;
4. The management strategies participants recommend for teething;
5. Participants’ awareness of professional resources and guidance available relating to teething; and the
6. Participants’ perception on the need for further guidance on the management of teething

## Method

This was a self-administered questionnaire-based study where participation was voluntary. *Ethical approval was not required for this study according to guidance published by the University of Cardiff. No formal application for ethics review was required as responding to the questionnaire was not considered to carry any form of risk or harm to the participants*.

### Participants

Specialists, Consultants and Specialist Trainees in Paediatric Dentistry in the UK who were members of the British Society of Paediatric Dentistry (BSPD) in January 2019 were invited to participate.

### Questionnaire design

Following a review of the literature to identify gaps in knowledge, an initial questionnaire was piloted with a small group of Specialists and Trainees (*n* = 5) and changes were made as a result of feedback. The final questionnaire consisted of a 10-item online survey including free-text questions presented using Microsoft Forms (Appendix 1), The survey consisted of five sections: participants demographics and professional background (five items); undergraduate education of the teething process (one item); awareness of teething-related signs and symptoms using a tick list of commonly reported symptoms (one item); the frequency and the management approach that is usually used for teething symptoms (two items); participants’ awareness of professional resource guidance available relating to teething (one item); and perceived need for further guidance on teething management (one item).

### Participant recruitment

A convenience, non-probability sampling method was used. No prior sample size calculation was conducted as the target population was small in number and therefore it was considered necessary to contact all members.

### Questionnaire administration

The BSPD agreed to administer this survey as it fell within their area of interest by allowing access to their membership lists. The BSPD administrator was sent the survey invitation and link. They distributed it through their membership lists (for Specialists, Consultants and trainees in Paediatric Dentistry) in January 2019.

The invitation email to participants included the questionnaire link that allowed them to complete the online survey. A reminder email was sent to all invitees one month later. As the survey was fully anonymous, non-responders were not followed up.

### Data analysis

Participants’ responses were transcribed into Microsoft Excel (Microsoft Corporation, Redmond, Washington). Descriptive data were presented as percentages and frequencies and the numbers were rounded to closest whole number. Cross tabulation was used to display the relationship amongst participants responses.

Responses to the open-ended question were viewed separately and themes were identified with no further analyses undertaken.

## Results

### Participant characteristics

From the 293 individuals who were invited to participate by email, 80 responses were received (27%). Most respondents were based in England (*n* = 58, 73%) and acquired their BDS degree between 2001–2010 (see Table 1)

**Table 1:** Participant characteristics (*n* = 80).

Participants details		*n* (%)
Year of BDS qualification	1971–1980	4 (5)
	1981–1990	10 (13)
	1991–2000	13 (16)
	2001–2010	**29 (36)**
	2011–2020	23 (29)
	No answer	1 (1)
Location at time of survey	England	**58 (73)**
	Wales	9 (11)
	Scotland	9 (11)
	Northern Ireland	4 (5)
Place of work	Dental Hospital (DH)	**55 (69)**
	Community Dental Service (CDS)	11 (14)
	Private practice	1 (1)
Level of training	Consultants	**33 (41)**
	Specialists	16 (20)
	Pre-Certificate of Completion of Specialist Training (Pre-CCST)	21 (26)
	Post-CCST	5 (6)
	Staff grade practitioners in Paediatric Dentistry	2 (3)
	No answer	2 (3)
	Other	1 (1)

Bold text implies the category with the highest responses.

### Participants’ undergraduate education about teething

As shown in Table 1, 76% (*n* = 61) of participants could not recall teething being taught in their undergraduate programmes, 16% (*n* = 13) recalled it being taught and 8% (*n* = 6) were unsure.

### Signs and symptoms attributed to teething

**Fig. 1:**
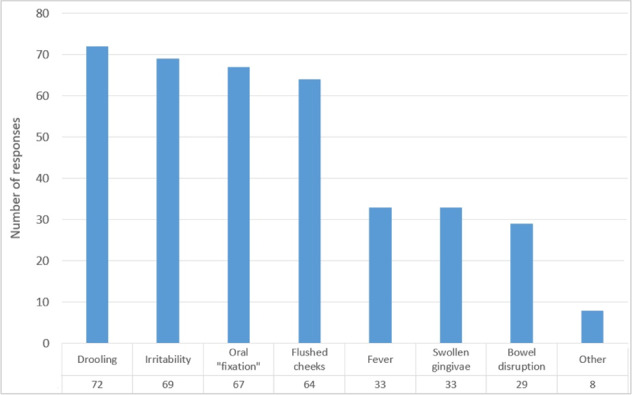
Frequency of teething signs and symptoms reported by the participants (*n* = 80). Participants could record more than one sign/symptom.

The signs and symptoms considered to be most commonly attributed to teething are shown in Fig. 1. The majority of participants thought that drooling (*n* = 72, 90%), irritability (*n* = 69, 86%), oral “fixation” (*n* = 67, 84%) were usually present when the child is teething. Only around one third of participants listed fever, swollen gingivae (*n* = 33, 41% for each) and bowel disruption (*n* = 29, 36%) as attributed to teeth eruption.

A few participants added further symptoms in the free-text comments: nappy rash (*n* = 2, 3%), sleep disturbance (*n* = 1, 1%) and pain (*n* = 1, 1%). Some respondents indicated their knowledge of symptoms was due to personal experiences as care givers, rather than as professional PDs.

### Participants’ experience of managing or providing advice

**Fig. 2:**
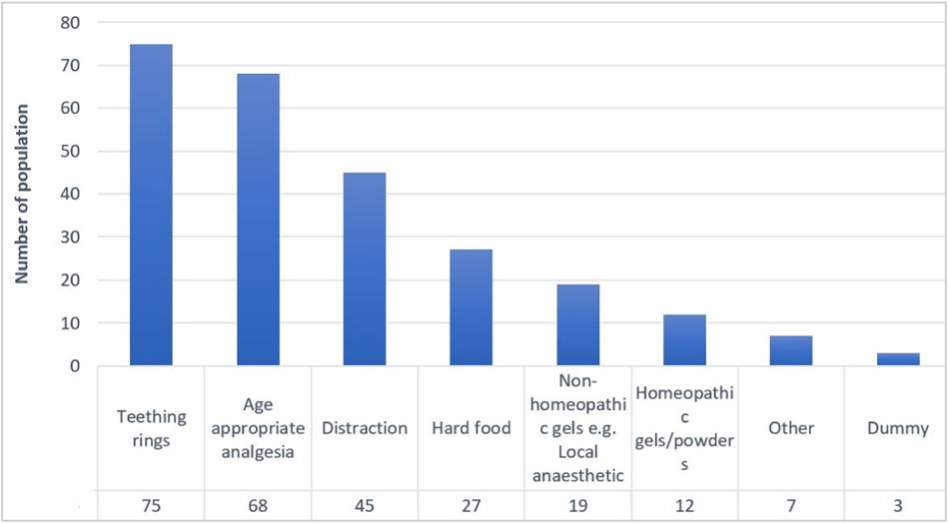
Frequency of reporting for management approaches of teething symptoms by participants (*n* = 80). Participants could record more than one management strategy.

Most participants reported giving advice on teething monthly (*n* = 35, 44%), followed by annually (*n* = 21, 26%) (Table 2). Figure 2 shows the frequency of the management strategies that they recommended using for teething. Giving the child a teething ring was the most popular management approach (*n* = 75, 94%). This is followed by recommending age-appropriate analgesia (*n* = 68, 85%). The free-text comments included clarification of occasional use of systemic analgesia and the use of cooled teething rings and foods. One participant advised that a differential diagnosis of other possible conditions would be needed and “possibly a mild sedative in consultation with a medical doctor.”

**Table 2:** Number and percentage of the participants’ responses (*n* = 80).

Participants details		*n* (%)
Participants’ undergraduate education	Yes	13 (16)
	No	**61 (76)**
	Not sure	6 (8)
Frequency of managing or providing advice	Daily	1 (1)
	Weekly	7 (9)
	Monthly	**35 (44)**
	Annually	21 (26)
	Never	3 (4)
	Other	11 (14)
Participants’ awareness of professional resources	None	**38 (48)**
	NICE CK	7 (9)
	MHRA	1 (1)
	BSPD	2 (3)
	NHS website	2 (3)
	Midwife/Health visitor/other professionals	2 (3)
	Other	8 (10)
Need for further guidance	Yes	**53 (66)**
	No	17 (21)
	Not sure	10 (13)

Bold text implies the category with the highest responses.

### Participants’ awareness of professional resources and guidance available relating to teething

Almost half of the participants (48%; *n* = 38) did not know guidelines were available. Seven of those who were aware of guidance mentioned the NICE CKS guidance. A further three participants (4%) reported a NHS website as another guidance source. Two participants (3%) stated incorrectly that there was guidance provided by the British Association of Paediatric Dentistry (BAPD) on teething. As for the perceived need for further guidance, many of the participants (66%; *n* = 53) were of the opinion that more guidance may be needed to manage the teething manifestations (See Table 2).

The comments from 20 participants who completed this section, were grouped into four themes (for example, see Table 3). These themes reflected some of the participants thoughts on the need for more patient’s education on teething and believing that management of the teething is not an exclusively dental issue and hence a more inclusive guidance that target other medical professionals is needed. Some participants chose to use the free-text comments to share their disbelief in teething as a condition and that it required a medical management.

**Table 3:** Free-text comments grouped by theme.

Need for further information for parents/carers	Need for collaboration with other health professionals	Concerns over “teething” as a condition	Concerns over “teething” as a condition
“A patient information leaflet and awareness would be useful”	“Often collaborative with Health Visitors and feel it would have more value if we presented the same information for teething and weaning.”	“I don’t believe in teething”	“I get very worried when parents give their children amber beads as a teething treatment due to the choking hazard. It seems this is quite a common practice.”
“Guidance needs to be aimed at parents more than dental professionals”	“I don’t feel teething guidance is required or relevant to specialist level – this is the kind of advice that would be most commonly given in primary care settings by health visitors or perhaps the GDP.”	“I think teething is just “normal” and usually associated with other minor illnesses – e.g. common virus.”	“… I don’t feel comfortable recommending homoeopathic/non-homeopathic remedies as I don’t know the science behind them or the safety aspects.”
“Parent information leaflets aspects.” would be handy”	“… My husband, as a GP, is inundated with questions. Teething is something that gets blamed for everything by the general public, child minders and health visitors and awareness of evidence and guidelines is low amongst new parents and child minders/ nurseries.”		

## Discussion

Signs and symptoms attributed to “teething” are reported by parents as being very common, yet this survey found that although PDs were consulted about it regularly, this was not frequent (at most on a monthly basis, for almost half of respondents). This indicates that they are either not the first source of information for parents/carers, or other allied health care professionals (AHPs) were consulted, or that parents/carers do not tend to seek advice.

One of the limitations of this study is that, although this study accessed the vast majority of PDs by using BSPD mailing lists, it likely missed out accessing some private PDs who were not members of the society. These are likely to be small in number so probably did not affect the results. However, the response rate (27%) was low, and this potentially limits the generalisability of the results. The responses given regarding signs and symptoms, aligned with most other studies. The survey respondents had broad representation, including PDs who ranged up to 45 years since BDS qualification (range: 1975–2017) and across the spectrum of training, from experienced PD Consultants to practitioners who mainly treat children with no formal postgraduate training. Most were located in England which aligns with UK workforce distribution [21].

Despite the potentially limited generalisability of this study due to the participation rate there is evidence to suggest that there is a broad church of views on the existence of teething, advice that is given when requested and on awareness of teething guidance. There was a significant level of support for development of better guidance. This study is part of efforts to develop robust and comprehensive, evidence-based guidelines on teething advice for both health professionals and parents [20]. Such guidance could help parents and professionals make accurate differentiation and diagnoses on whether children are “teething” or have another illness. It will also inform advice and management in response to teething symptoms, driving these towards evidence-based options rather than resorting to medicalisation with over-the-counter teething products as a first line of treatment, especially as some of these contain ingredient which are potentially harmful for children [20].

Most signs and symptoms that respondents attributed to teething were localised manifestations such as drooling and irritability. Some PDs felt that teething was not associated with systemic illness. These views agree with research [7, 8] where an increase body temperature might be found but that fever is not associated with teething [9], and hence, further medical attention should be considered for babies and children with fevers. The views of PDs diverged from some that have been previous reported for similar groups of clinicians in the literature, in terms of associating fever with teething [13]. This might be due to differences in the cultural and clinical backgrounds. These discrepancies highlight how beliefs around childhood teething can be contextually related.

There was general agreement amongst PDs on recommending chewing a soft teething ring or using age-appropriate analgesia as first-line management strategies for teething symptoms. These non-invasive approaches, to treat mild signs and symptoms, are recommended by guidelines [11, 22]. However, around 30 of the PDs had no awareness on guidelines for teething.

At the time of the survey administration, there were three sources of advice on teething accessible to professionals in the UK. The Scottish Dental Clinical Effectiveness Programme’s Management of Acute Dental Problems [23] a reputable, evidence-based guideline. It mentions pain around “a newly erupted tooth” but does not mention teething specifically. The advice given concerns oral hygiene and self-care measures. The American Academy of Paediatric Dentistry guideline on Perinatal and Infant Oral Health Care recommend oral analgesia and chilled teething rings as well as avoiding topical anaesthetic gels [22] The NICE CKS summary of available evidence goes into more detailed recommendations, advising reassurance, basic oral hygiene advice and encouragement to seek dental care in the first instance [11]. However, there is no specific guidance relating to the management of teething symptoms. Respondents felt that advice regarding the management of teething should be developed for, and accessible to, primary dental care providers and other AHPs and there is evidence that providing parent/carers with information on managing teething symptoms can reduce medication use [24].

Simply producing guidance is not enough to make the target audiences, aware of it, and even when they are aware of it, it does not mean it will be adopted. Alongside producing guidance, barriers to the uptake of recommendations in everyday practice and use of strategies to overcome these in dissemination should be considered. Perhaps one of these barriers is that some PDs, as revealed in their comments, did not consider “teething” to be a condition in its own right. The existence of “teething”, and the degree to which the signs and symptoms commonly attributed to it actually are caused by tooth eruption, is controversial and an area that needs to be resolved [25, 26]. This gap in knowledge is currently filled with culture-inspired remedies and commercially available, OTC products that promise parents relief of their child’s discomfort, without the need to seek a professional opinion.

PDs are ideally placed to provide appropriate advice and reassurance to parents and are also as an expert source of information for other AHPs, such as Health Visitors, who are in regular contact with parents of teething infants. Up-to-date, accessible guidance should be available to PDs and for them to refer other AHPs.

Another limitation of the study was associated with the lack of a pre-existing questionnaire tool that could be adopted. The one we used was not formally assessed for its validity and reliability. Hence, the study findings need to be considered with caution. However, the content validity for the questionnaire was assessed through questionnaire piloting with a similar sample to the population of interest. Their feedback was incorporated into the final version of the questionnaire.

## Conclusion

Signs and symptoms of teething might mask underlying conditions so teething should be a diagnosis of exclusion. PDs attributed similar signs and symptoms to teething as have been reported in the literature, although some commented that they did not think teething was a condition. There was a lack of awareness over existing guidelines on teething, although these guidelines do not offer clear information to guide decision-making. Creation and wide dissemination of guidelines on teething is recommended.

## Supplementary information

Appendix 1 Questionnaire for perception of teething
