# Supplementary material for: A questionnaire-based study of Paediatric Dentists’ knowledge of teething signs, symptoms and management
Source: BDJ Open. 2022 Mar 12;8:7. doi: 10.1038/s41405-022-00099-4 (PMC8918333; doi:10.1038/s41405-022-00099-4)
Supplement: Supplementary file 1 — Appendix 1 Questionnaire for perception of teething [file 41405_2022_99_MOESM1_ESM.pdf]

Appendix 1. Questionnaire form

Paediatric dentists knowledge on teething

Please find the participant information sheet here – http://tinyurl.com/y4g2f363

1. Typically who often do you give advice on teething?
   a. Daily
   b. Weekly
   c. Monthly
   d. Annually
   e. Never
   f. Other ……….

2. In your experience what symptoms do you feel are attributed to teething? (you may select more than one option)
   a. Fever
   b. Drooling
   c. Irritability
   d. Flushed cheeks
   e. Swollen gingivae
   f. Bowel disruption
   g. Oral "fixation" or chewing objects
   h. Other ………….

3. What guidance in relation to teething are you aware of?

4. Do you feel that there is a need for better guidance on the management of teething?
   a. Yes
   b. No
   c. Unsure

5. Which of the following would you advise for the management of teething symptoms?
   a. Distraction/comfort
   b. Use of a dummy
   c. Teething rings
   d. Hard food to chew
   e. Homeopathic teething gels/powders, e.g. Ashton and Parsons powder, Nelsons Teetha gel and granules
   f. Non-homeopathic teething gels/powders, e.g. those containing a local anaesthetic

g. Age appropriate dosage of oral systemic analgesia, e.g. paracetamol or ibuprofen
h. Other ……………

6. Which area of the UK do you primarily work in?
   a. England
   b. Northern Ireland
   c. Scotland
   d. Wales
   e. Other…………..

7. What setting best describes your primary work place
   a. Private practice
   b. Community Dental Service (CDS)
   c. Hospital unit (excluding dental hospital)
   d. Dental hospital
   e. Other ……………

8. Which of the following best applies to your role?
   a. Specialist in Paediatric Dentistry
   b. Consultant in Paediatric Dentistry
   c. Pre-CCST trainee in Paediatric Dentistry
   d. Post-CCST trainee in Paediatric Dentistry
   e. Staff grade in Paediatric Dentistry
   f. Other ……………..

9. In what year did you qualify with your BDS?

10. Do you recall the management of teething being taught during your undergraduate BDS degree?
    a. Yes
    b. No
    c. Unsure

11. Any other comments
